# It’s a Long Way to the Tap: Microbiome and DNA-Based Omics at the Core of Drinking Water Quality

**DOI:** 10.3390/ijerph19137940

**Published:** 2022-06-28

**Authors:** Antonia Bruno, Giulia Agostinetto, Sara Fumagalli, Giulia Ghisleni, Anna Sandionigi

**Affiliations:** 1 Biotechnology and Biosciences Department, University of Milano-Bicocca, 20126 Milan, Italy; giulia.agostinetto@unimib.it (G.A.); sara.fumagalli@unimib.it (S.F.); g.ghisleni2@campus.unimib.it (G.G.); 2 Institut Jacques Monod, Université Paris Cité, CNRS, 75013 Paris, France; 3 Quantia Consulting S.r.l., 22066 Milan, Italy; anna.sandionigi@quantiaconsulting.com

**Keywords:** drinking water, microbiome, omics

## Abstract

Microbial communities interact with us and affect our health in ways that are only beginning to be understood. Microorganisms have been detected in every ecosystem on Earth, as well as in any built environment that has been investigated. Drinking water sources, drinking water treatment plants and distribution systems provide peculiar microbial ecological niches, dismantling the belief of the “biological simplicity” of drinking water. Nevertheless, drinking water microbiomes are understudied compared to other microbiomes. Recent DNA sequencing and meta-omics advancements allow a deeper understanding of drinking water microbiota. Thus, moving beyond the limits of day-to-day testing for specific pathogenic microbes, new approaches aim at predicting microbiome changes driven by disturbances at the macro-scale and overtime. This will foster an effective and proactive management of water sources, improving the drinking water supply system and the monitoring activities to lower public health risk. Here, we want to give a new angle on drinking water microbiome research. Starting from a selection of 231 scientific publications on this topic, we emphasize the value of biodiversity in drinking water ecosystems and how it can be related with industrialization. We then discuss how microbiome research can support sustainable drinking water management, encouraging collaborations across sectors and involving the society through responsible research and innovation.

## 1. Introduction

Drinking water quality is of direct relevance to human health because it is the primary source of human sustenance. Drinking water quality reflects the characteristics of raw water source, both from the physico-chemical and the microbiological point of view.

Often, a raw water source requires treatments before being considered potable. Furthermore, drinking water is delivered to the consumer through kilometers of pipes, and maintenance of water quality in these long ways to the tap is a prime concern for drinking water utilities.

Thus, providing safe and high-quality potable water can be a challenging task.

According to the Revised Drinking Water Directive (2020/2184), a total of 48 microbiological, chemical and indicator parameters must be monitored and tested regularly. However, considering specifically microbial safety, the focus is mainly on testing for the presence of bacteria indicating fecal contamination. These are measured using well-established culture-based tests, but with clear limits: they investigate for only specific microorganisms, without taking into account the wide range of microorganisms that constitute drinking water; moreover, those recalcitrant to growth in laboratory conditions are missed.

The assemblage of microbes within drinking water is referred to as the drinking water microbiota (“microbiome” when referring to the associated genetic information), and it accounts for about 10^6^–10^8^ cells/L [1].

In this context, exploring the whole microbial community of an ecosystem and its interactions with biotic and abiotic factors is pivotal. Advances in DNA sequencing technologies and computational analyses are fostering the application of DNA-based omics for understanding microbial community structure and dynamics to preserve and improve drinking water quality. High-throughput DNA sequencing (HTS) technologies (also called next generation sequencing technologies, NGS) are able to massively sequence millions or billions of DNA sequences with high sensitivity. This allows not only the detection of even DNA traces, but also the generation of an unprecedented amount of data, especially in the case of complex matrices (in terms of biodiversity), such as water. The inconceivable rise of omic tools has turned the exploration of such a peculiar ecosystem within reach.

Indeed, we interrogated the ISI Web of Science database on 29 May 2022 to collect the recent studies published on the topic of drinking water microbiome. We selected the period starting from 2013, when HTS techniques started to be applied, and we used the following query:

“ALL = (“drinking water” microbiome) OR ALL = (“drinking water” microbiota) OR ALL = (“drinking water” metagenomics)”.

We obtained 1113 hits. Then, we manually refined the research, excluding the scientific publications not pertaining to the subject, down to a selection of 213 publications (Supplementary Table S1).

**Figure 1:**
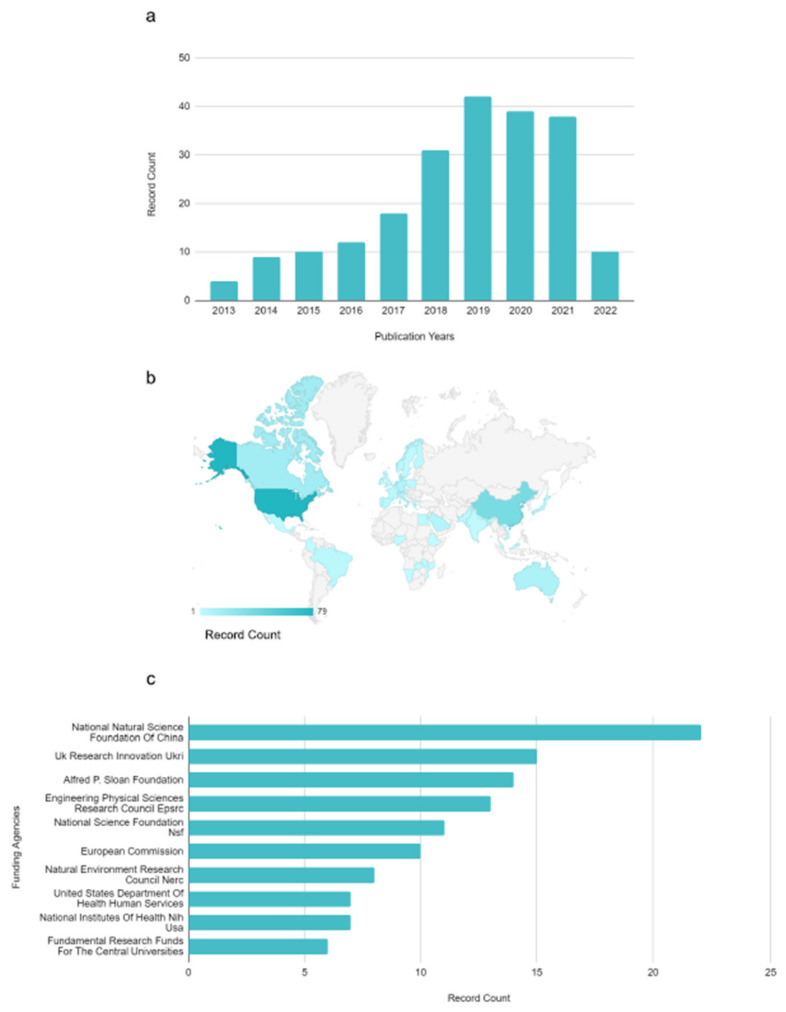
Published papers from 2013 concerning drinking water microbiome, obtained from ISI Web Of Science database. (**a**) Bar chart distribution of published papers by year. (**b**) Geo chart showing the distribution of published papers per country. (**c**) Top ten funding agencies contributing to published papers. Record count: published papers, *n* = 213.

An exponential growth in publications regarding this topic clearly appears (Figure 1a). Surprisingly, microbiome studies focused specifically on drinking water microbiome are still fragmentary, although the application of microbial ecology supported by HTS analyses is promising in drinking water management. For instance, most of the studies are concentrated in the US, China and Europe, with less coverage in the other countries (Figure 1b). This could be explained by looking at the funding agencies which more supported the scientific publications examined, mainly located in China, US, and Europe (Figure 1c).

Starting from this selection of 231 scientific publications, we emphasize the value of biodiversity in drinking water ecosystems and how it can be related with industrialization. We then discuss how microbiome research can support the sustainable drinking water management of drinking water quality, encouraging collaborations across sectors and involving the society through responsible research and innovation.

## 2. A Biodiversity Matter

Aquatic ecosystems are among the most biodiverse ecosystems on Earth, also looking at microscopic life. Drinking water makes no exception, showing an astounding biodiversity in terms of microorganisms harbored [2]. Indeed, the term “diversity” appears among the most abundant words (count = 111) of the abstracts selected by the research in the ISI Web of Science database (Supplementary Figure S1).

It is clear that the concept of biodiversity changed consequently to omics advances. Pioneering microbiome projects (e.g., Human Microbiome Project [3], Earth Microbiome Project [4], and many others) gave a boost in protocols development and DNA sequences acquisition, so that it is now possible to explore the biodiversity of an environmental sample in a time- and cost-effective manner.

In particular, the use of DNA metabarcoding applied to suitable molecular markers (i.e., 16S rRNA hypervariable regions) was fully utilized in very different contexts, gaining high standard experimental protocols and cheap services.

Considering drinking water microbiome research, a recent meta-analysis [5] of existing and available Illumina 16S rRNA datasets from drinking water source, drinking water treatment (DWTP) and drinking water distribution systems (DWDS) calculated a total of 22,754 unique taxa. This impressive diversity in terms of bacterial DNA sequences has been tracked from source to the tap: the highest degree of sequence diversity in terms of richness and Shannon index values came from untreated water, but a reduction in these values was evident in DWTP and DWDS groups.

Thus, what happens to the drinking water microbial community along the journey from the source, through the potabilization processes and distribution systems, to the tap?

### 2.1. Microbial Biodiversity in DWTP and DWDS

Groundwater and surface water are the two main types of source water to provide drinking water to the population. They have intrinsically different physical and chemical characteristics: groundwater are dark, oligotrophic environments, mostly known to be anoxic or anaerobic. The presence of iron, manganese, ammonia, sulfur compounds, methane, and dissolved organic carbon supports the growth of anaerobic communities without light sources [6,7,8]. On the other hand, surface waters are more affected by seasonal and environmental factors, such as weather and temperature, and thus, the concentration of certain chemicals is more variable. Being groundwater and surface water ecosystems with different characteristics, the microbial communities are also different [9]. For instance, groundwater has been shown to harbor microorganisms belonging to the Candidate Phyla Radiation (CPR) and even ultra-small bacteria (see Section 2.2) [10,11]. Conversely, surface waters seem to be not dominated by these uncultivable, new-to-science microorganisms, but they show the presence of Cyanobacteria. Actinobacteria, Bacteroidetes, and Proteobacteria are shared by the two different water sources [12].

These microbial populations can seed downstream microbiota in the DWTP. The review of Zhou and colleagues exhaustively summarized the changes in the bacterial community and the influence of each treatment stage on microbial diversity in full-scale water supply systems [12].

Actually, potabilization processes involved in DWTPs are based on physical and chemical treatments to remove unwanted chemicals and microorganisms, such as coagulation and sedimentation, filtration through sand filters, granular activated carbon filters (GACs), and biological activated carbon filters, and finally disinfection. It is worthy to note that the seminal paper of Pinto and colleagues first shed light on the role of filtration in microbial community assembly in DWTP: carbon filters harbor a microbial community that can seed the downstream water [2,13]. Indeed, bacteria defined as “leaky colonizers” (e.g., *Hydrogenophaga*, *Acidovorax* and *Denitratisoma*) can colonize GAC/sand filters and can shape the drinking water microbiome downstream. Some studies also revealed the predominance of Bradyrhizobiaceae family and the enrichment in bacteria-carrying functions associated with aromatics degradation, many of which were encoded by Rhizobiales, in granular activated carbon filters [14]. Filter-specific occurrences have also been reported [14,15,16]. Disinfection through chlorination, chloramination, and UV can be employed to reduce the bacterial load. Generally, after disinfection, Alpha- and Beta-Proteobacteria are reported to be dominant in chlorinated water, whereas Beta-Proteobacteria are more abundant after chloramine disinfection than the other two processes [12].

Defining a conserved group of taxa characterizing drinking water exiting DWTP can be difficult, due to the variables related to water source and treatments mentioned above. However, at higher taxonomic levels, such as phyla and classes, a core microbiome constituted by Alpha- and Beta-Proteobacteria, and to a lesser extent of Gamma-Proteobacteria, Nitrospirae, Planctomycetes, Acidobacteria, Bacteroidetes and Chloroflexi, can be identified. At lower taxonomic level, Burkholderiaceae, Methylophilaceae, Comamonadaceae, and Rhodocyclaceae were abundant among Beta-Proteobacteria, whereas Sphingomonadaceae, Caulobacteraceae, and Methylobacteriaceae were dominant in Alpha-Proteobacteria [17].

The ecological dynamics occurring along the DWDS are well described in the review of Douterelo and colleagues [18]. Especially in DWDS, in addition to existing in a planktonic state (i.e., in the bulk-water), microorganisms can form a biofilm (eventually resistant to disinfectant) adhering to the pipe surfaces [19]. Biofilms are clusters of microorganisms that stick to nonbiological surfaces, such as rocks in a stream or pipes, as well as living forms (host-associated). Biofilm formation is spread in nature and serves as a defensive mechanism, produced by the microorganisms themselves or the host [20]. Resolving drinking water biofilms is a key aspect: biofilm formation can be easily seen in DWTPs and DWDSs because it represents a protected mode of growth that not only allows cells to survive in hostile environments but also to colonize new niches by dispersal of microorganisms from the microbial clusters [21]. After leaving DWTP, water reaches consumers after hours or days, depending on the system hydraulic retention time, a function of supply demands and network distances. A greater contact time with the DWDS infrastructure is likely to accelerate water quality degradation, and pipe material, dimension, and structure can affect biofilm formation [21,22]. Clearly, DWDS hosts extensive microbiomes with diverse biofilm communities, depending on several factors, such as flux and other hydraulic variables, but also the interaction with other microorganisms. A core microbiome is hard to be identified, considering differences in potabilization treatments, operational practices, and distribution system characteristics [5,23].

The meta-analyses conducted by Thom and colleagues [5] resolved the core microbiome of DWDS and proposed as prevalent taxa *Sphingomonas* (Alpha-Proteobacteria), an uncultured Rubinsphaeraceae (Planctomycetes), and *Hyphomicrobium* (Alpha-Proteobacteria) in DWDS bulk water and *Sphingomonas*, an uncultured Rubinsphaeraceae, and *Pseudomonas* (Alpha-Proteobacteria) in DWDS biofilm.

On the whole, the 25 most abundant genera considering the entire flow from source to the tap identified in this meta-analysis included organisms that are not usually tested for drinking water contamination but may have a role in drinking water quality. For instance, there were no coliforms identified in the core microbiome analysis or taxa profile for any meta-sample groups. It is worth noting that the most common genera in DWDS were less abundant in source and treatment, except *Nitrospira*, which was more abundant throughout treatment than in DWDS.

If many studies take a snapshot of specific points along the flow from the source to the tap and of specific moments, several works evaluate the microbiome considering its dynamics in space and time. For instance, omics strategies helped in disentangling spatial–temporal bacterial community in DWDSs in Northern China [24], exploring the UV/chlorine disinfection for the monitoring of potential opportunistic pathogens and the evaluation of risks caused by transregional water diversion to local water. Similar studies were conducted in Valencia [23], Paris [25], Milan [2], Zurich [15], more than one city in The Netherlands [26], Ann Arbor (US) [13], Urbana-Champaign (at the University of Illinois) [22], and many other.

On the whole, more and more data suggest that water treatments (in particular chlorination) significantly reduce overall species abundance and richness. Conversely, more stochastic processes governed the assembly of microbial communities in DWDS biofilm [5].

Beside the taxonomy characterization, also understanding microbiome functional properties is important to determine the behavior of a microbial community, mostly considering a predictive point of view. In this sense, shotgun metagenomics has revolutionized the way to study microbial dynamics, adding the information of functions. In this way, we can explore not only who are the actors (as in 16S rRNA sequencing approach), but also what they are doing. Moreover, shotgun metagenomics allows the monitoring and investigation of antibiotic resistance spread, an issue that is gaining increasing importance, considering the selective pressure that may be applied by potabilization processes [27,28]. Metagenomics-based approaches could also be used to identify “nonfunctional” microorganisms that play a key role in maintaining the stability of the whole microbial ecosystem. They underpin ecosystem functions, playing a crucial role in maintaining ecosystem equilibrium and health.

Although the effect of some microorganisms on human health are not well characterized, variations in microbial biodiversity and subsequently the bioactivity may affect the resilience of all other organisms and hence their ability to respond to anthropogenic pressure [29].

### 2.2. Microbial Dark Matter: Gap of Knowledge about the Uncultivable Majority

Considering the astonishing biodiversity that is emerging from microbiome studies, it would not be surprising if many of the microorganisms detected are unknown or poorly characterized. This also relies on the so-called Great Plate Count Anomaly [30]: in many environments, only 0.1–1% of microorganisms have been cultivated because of the difficulties to recreate in laboratory conditions the real environmental characteristics that allow the growth.

Nevertheless, unknown microbial life may be playing a crucial and even dominant role in ecosystem equilibrium. Borrowing the terms from astronomy, microbiologists have defined these still unknown microorganisms as “microbial dark matter” because they likely account for a large portion of the Earth’s biomass and biodiversity, yet their basic metabolic and ecological properties are not known. Phyla composed exclusively of uncultured representatives are referred to as Candidate Phyla (CP) [31,32,33].

The candidate bacterial superphylum Patescibacteria (also known as Candidate Phyla Radiation, CPR) and archaeal superphylum DPANN (Diapherotrites, Parvarchaeota, Aenigmarchaeota, Nanoarchaeota, and Nanohaloarchaeota) [34,35,36] are some of the uncultivable microorganisms uncovered in water ecosystems using high-throughput DNA sequencing techniques. We can surely affirm that the application of culture-independent methods has broadened our view of the tree of life [11,36,37], especially considering microorganisms which live in poorly understood environments as groundwaters. Due to their restricted metabolic capacity and unusual biology [10,11,35], it was suggested that Patescibacteria and DPANN are probably in a syntrophic relationship with other microorganisms, relying on them for complete redox transformations. The intricate pattern of dependencies between these microorganisms may explain the difficulty of their growth in laboratory conditions.

Patescibacteria are predominant in groundwater probably due to their thriving in oligotrophic environments and mobilization from soils [38]. Furthermore, recent studies [2,16] have found a high abundance of Patescibacteria in GACs as a possible consequence of groundwater seeding of carbon filters and filter morphology characteristics. The presence of microbial communities such as Patescibacteria influences the downstream water characteristics. Therefore, applying stressors such as potabilization treatments to these oligotrophic ecosystems can greatly affect the selected microbial communities and the water we drink.

The catchy definition “microbial dark matter” contains imprecisions if compared with the astronomical “dark matter” [31]. However, it can help enhance the interest for the neglected microbial biodiversity harbored by unconventional environments, such as drinking water ecosystems [7].

## 3. Living in a Built Environment: DWTPs and DWDSs Ecosystems

The built environment (BE) encompasses all the environments that humans have constructed, including buildings, cars, public transport, and other human-built spaces, as well as DWTPs and DWDSs [39,40].

The built environments harbor unique microbial communities, different from those found in other environments on Earth. Often these constructed habitats are designed to be harsh for life, probably setting the conditions for a selective pressure that drives the built environment microbial assemblage [41].

Natural and constructed habitats are interconnected, especially in the case of water resources treated for human consumption, where drinking water source is collected from natural (sometimes pristine) environments and enters a manmade ecosystem. The flow has a direction from raw water upstream to treated water at the tap, and along this flow, multiple stressors can act on the microbial communities and their structures.

Microbial assemblage dynamics can be governed by both deterministic and stochastic processes in natural and built environments [42,43]. If deterministic factors are niche-based factors which can be controlled and manipulated, the effects of stochastic factors (such as genetic mutation, gene duplication, cell damage by radicals, die-off, interspecies interactions, emigration, immigration and random drift) on microbial assemblage are difficult to predict. For these reasons, DWTPs and DWDSs, as built environments, must be monitored and controlled taking into account those ecological processes that can relate to stochasticity in microbial assemblages [42]. For instance, opportunistic pathogens, such as *Legionella, Mycobacterium,* and *Pseudomonas*, find a favorable ecological niche in premise plumbings that are in effect a front line of human exposure [44].

Looking at a DWTP, microorganisms from drinking water source (groundwater and surface water) seed water downstream [13], colonizing the treatment units, such as granular activated carbon filters [2,16], surfaces and pipes [21,45], and can be released to subsequent processes and then to the DWDS. This directional movement is called migration (or immigration) and significantly contributes to microbial assemblage: in the neutral theory of biodiversity and biogeography, immigration is one of the key stochastic processes that change the community assemblage [46]. Moreover, water stagnation, intermittent supply, and the bi-directional migration from pipes to water and vice versa in the distribution network and indoor plumbing [22,47] must be taken into account. Mei and Liu [48] addressed the crucial question about to what extent immigration contributes to the assembly and function of the downstream community in DWTPs and DWDSs. What clearly appears is that commonly used methods, mainly based on culturing bacterial indicators and measuring chemical parameters, are not enough to quantitatively determine the microorganisms carrying out the process: to deeply understand these dynamics, an ecogenomics-based mass balance approach is proposed, an approach that couples a mass balance model with high-throughput DNA sequencing. Moreover, it can be coupled with machine learning to improve environmental variables identification [48].

Thus, monitoring, when stochasticity exists, is a technological challenge. De Vrieze and colleagues [42] proposed to first depict the initial status of the microbial communities, then to evaluate the deterministic processes (potentially) influencing the microbial community, and the (change in) input streams. The degree of microbial community dynamics, i.e., the change in microbial community composition in function of time, can be obtained on the genetic level through HTS-based microbiome analyses.

Linked to this, the estimation of the biological stability of drinking water, by the application of DNA-based omic technologies and powerful bioinformatic approaches, is a crucial node. Gomez-Alvarez and Revetta [49] demonstrated the potential of water microbiome profiles coupled with machine learning analyses as bioindicators for system stability in DWDS. This paves the way for the use of indicators that are microbiome-based: data-driven approaches can improve the predictive ability of the models applied to prevent risks, when classical bioindicators fail.

## 4. Microbial Exposure and Industrialization: How Are They Linked with Drinking Water?

Urbanization and the westernized lifestyle have heavily affected human health. Over the last decades we have witnessed a significant increase in allergies and multifactorial inflammation-related chronic diseases diagnosis rate [50]. An inspiring review by Sonnenburg and Sonnenburg [51] highlighted the vulnerability of the “industrialized microbiota”: more than other factors, sanitation, industrial advances and diet have shown to have a role in the fragility of human microbiota, and as a consequence in human health.

Indeed, our current and highly hygienic way of life—thanks to which we avoid the spread and the development of the infective diseases that caused numerous deaths in the past—caused a mismatch between these environmental conditions and the past high pathogens exposure under which the immune system evolved. The so-called Hygiene Hypothesis seems to have found an explanation for the considerable spread of asthma, eczema, rhinitis, food allergies, but also for type 1 diabetes, and inflammatory bowel diseases [52]. It is worth noting that the study of Von Hertzen and colleagues [53] correlated microbial-rich drinking water with reduced risk for atopy. The Hygiene Hypothesis principle, together with the recent increasing interest in the study of microbiota, lead to the development of the Old Friends Hypothesis [52]. This describes how some mechanisms of immunomodulation are encoded in the genes of organisms that coevolved with humans; thus, the interaction of the immune system with these organisms is essential for an appropriate immune response. These evolutionary considerations are conducive to important medical implications (for instance helminth immunoregulation therapies [54,55]) and to the discovery that environmental microbiota exposure of the mother throughout pregnancy and of the child during the early life can train the immune system not to over-react to future exposures and shape the symbiotic host-associated community composition starting with the first “pioneer microbes” [50]. A new definition of hygiene that considers the beneficial effects of the symbiotic relationship we have with certain organisms, without excluding the negative effects pathogens have on our health, has indeed become necessary. Coherently with the “raw water trend” discussed in Section 6, standard treatment practices should aim to limit the spread of pathogens instead of removing all microbiota from drinking water [56].

Blum and colleagues [57] proposed the existence of a close linkage between soil and human gut microbiota. Since the hunter–gatherer origin of our societies, the human activities, food resources (including water) and habitations have always been strictly dependent on the soil we live on; furthermore, even today, especially during early childhood, we ingest and inhale microorganisms from the environment and in particular from the soil. It is known that animals can acquire part of their microbiota horizontally from the environment [58], and, for these reasons, we can find autochthonous and allochthonous organisms that together contribute to the dynamic composition of the gut microbiota. In support of the strong influence that soil has on the gut microbiota composition and of the Old Friends Hypothesis, we find the “Farm Effect”: children raised in traditional farm communities are less prone to developing asthma and allergies [50]. Similar to soil, we can argue that drinking water, being directly and intentionally ingested on a daily basis (and from the origin of our species), can be considered as a microbial reservoir and a source that affected and still affects the composition of the human gut microbiota. We cannot ignore that, especially in developing countries but also in developed ones, poor drinking-water quality and management are related to several life-threatening diseases (such as diarrhea, nematode infections, lymphatic filariasis, schistosomiasis) and that it is estimated that 10% of the global disease burden could be preventable by enhancements of drinking water quality and sanitation [59]. Water treatments (such as ultrafiltration) not only remove NOM (Natural Organic Matter) but also decrease the bacterial load [21], eliminating pathogens and non-pathogenic microorganisms. Safeguarding public health is imperative all over the word, and if the transition from well water to treated water consumption has considerably improved life conditions, on the other hand, it has created a vulnerable and invasions-prone context. Equally to what happens when sterilized systems are exposed to the environment, deficiencies in the treatment process can indeed make drinking water susceptible to invasion of opportunistic and potentially pathogenic organisms due to its low diversity and the availability of niches freed by the treatments [42]. Nontuberculous mycobacteria, responsible for pulmonary infections and *Legionella* sp., have been found in public water distribution systems and treated drinking water [60,61]. Moreover, global invasions of the drinking water system by the protozoan pathogens *Cryptosporidium* and *Giardia* species were recorded in the 1990s in Western countries [62] and are still common in developing countries [63]. These data stress out the extreme vulnerability of the fundamental drinking water system we rely on and suggest the advantages of metagenomic analysis in monitoring and detecting the drinking water microbiota composition.

Finally, trying to understand if there is a significant correlation among drinking water source, microbiota and health, the work of Vanhaecke and colleagues [64] evaluated the associations of drinking water source (bottled, tap, filtered, or well water) with global gut microbiota composition, starting from the data extracted from the American Gut Project public database. Even if they do not inspect drinking water microbiome, they demonstrated that drinking water source was associated with differences in gut microbiota signatures. Subjects drinking mostly well water had higher fecal α diversity, higher *Dorea*, and lower *Bacteroides*, *Odoribacter*, and *Streptococcus* than the other groups. Low water drinkers also exhibited gut microbiota differences compared with high water drinkers. This study strongly suggests the need to implement drinking water microbiome investigation to better understand the role of environmental microbes in health and disease.

The main question remains: “*How do we “conserve” beneficial bacteria and keep the pathogens at bay?”* [51].

## 5. Sustainable Development Goals (SDGs) 5 and 6 for a More Sustainable Future in Managing Drinking Water

Worldwide, 2.2 billion people do not have access to safely managed drinking water, with 785 million of them without basic drinking water [65]. In the future, the situation is predicted only to worsen with 8.5 billion people on this planet and 40% of freshwater supplies insufficient by 2030 [65]. Thus, the solution for our survival on Earth lies in developing sustainable and well-engineered systems to produce human services [42], such as water purification and sanitation.

The answer can be achieved from natural resources themselves: in particular, microorganisms can be the prompt to cope with sustainable development goals (SDGs) [66]. Ranging from the most foreseeable SDG6 (clean water and sanitation) to the less immediate SDG5 (gender equality), microorganisms can have a key role to provide innovative and alternative solutions. Instead of separated issues, gender equality and access to safe water and sanitation are deeply linked and interdependent [67]. In 80% of the cases, if water is not available on the premises, women and girls are responsible for fetching it with all the consequences that result (e.g., education loss, increase in abuse and physical injuries [68]), making gender equality and the right to clean water and sanitation strictly connected [67,68,69]. Increasing the availability and quality of sex-disaggregated data on water, sanitation and hygiene, and increasing leadership and participation of women in water governance are among the recommendations within gender-transformative water programmes. Thus, finding a sustainable solution for clean and safe water is key to improving lives, especially for women.

For centuries, microorganisms have played a crucial role in providing safe drinking water, renewable energy, and food products [42]. Therefore, microbiome-implemented processes will continue to contribute to human society, ranging from biodiversity, climate change, sustainability, and human rights. Microbial application to water management is a so-called nature-based solution (NBS), defined as “*living solutions inspired by, continuously supported by and using nature, which are designed to address various societal challenges in a resource-efficient and adaptable manner”* [70]. Indeed, water purification for human consumption is one of the ecosystem services that microorganisms drive [71], for example through the reduction in contaminants or pollution in water [72,73], allowing water reuse. Intuitively, its reuse can alleviate the pressure on primary water resources, such as surface and groundwaters, by providing a sustainable source of drinking water [74]. Moreover, water reuse provides qualitative benefits, as it reduces the UWWTP (urban wastewater treatment plant) discharge into the environment and relieves greenhouse gas emissions, requiring less energy than other water supply sources [74].

At the same time, it is well known that premise plumbing pathogens are responsible for infections and diseases [44]. Unfortunately, it is impossible to eliminate all risks associated with the water microbiota, due to the stochastic factors shaping microbial communities [42]. Anyway, by taking the necessary precautions and monitoring levels, e.g., using portable DNA sequencing devices such as MiniION^TM^ [75,76,77] and online monitoring [78], microbiome-based research will continue to pursue a sustainable future.

Improved water quality by microorganisms has clear implications for human health and well-being, and, with some lateral thinking, we can affirm that microbiology is relevant to all the SDGs [79]. The multidisciplinary nature of microbiological research will facilitate an integrated and sustainable approach, which, however, must rely on a robust and deep knowledge of the micro-scale processes and the macro-scale effects.

## 6. Building Responsible Research and Innovation

As long as we are reluctant about the great biodiversity of microscopic life all around us (without the exception of drinking water), ensuring sustainable and high-quality drinking water will be difficult to achieve.

The discovery of microorganisms dates back to the 17th century with Antonie van Leeuwenhoek, who designed a single-lens microscope which allowed him to see “animalcules”, tiny animals (which were instead bacteria), in a water sample [80,81]. Despite the scientific advancements and increased knowledge about microbial communities and water ecosystems, in our present society, the perception of microorganism presence in pure water is seen as harmful anyway. This perception is historically understandable, given the fact that pathogen microorganisms have killed more than any other agent. At the same time, a new phenomenon is rising, the so-called “raw water trend” [82]: the request for unfiltered, untreated, unsterilized water, suggesting that a return to a “raw” lifestyle would be healthier and safer, is worryingly spreading. We are clearly facing a failure in communication of science on one hand and on the other a misinterpreted right to express opinions and concretize actions that can have risky effects on public health. The “democratization of knowledge” should be aided with transparency and proper science communication tools; otherwise, it opens the way for caveats and pitfalls [83].

In this context, an increased consciousness, based on scientific studies coupled with outreach actions, of the necessity of drinking water monitoring and, when needed, treatment can help in defeating devious information.

One virtuous and unique example of a citizen science project aiming at increasing involvement of the society about the thematic of drinking water microbiome is the ‘Freshness of Water’ citizen science project [84]. In this project, citizens of Amsterdam participated in taking samples from their own kitchen tap and were involved in the analysis process and updated on the results. Interestingly, for the participants, the presence of tens of thousands of bacterial species in their drinking water, as well as the interpretation that this is perfectly normal and not a health issue, was not a concern, even if it was a completely new concept. This is because of the transparency used in communicating the results and the deep explanation even of complex concepts about microbial ecology. Indeed, the involvement of citizens from the beginning to the end of the projects, making them pro-active participants, functioned as a driving force to increase their confidence about drinking water quality. The majority of the citizen scientists state that, as a result of their participation, their confidence in the water company has also increased.

Clearly communicating the biological complexity of water, and the importance of research as a discovery process, will incentivize scientific advances and innovation, increasing levels of confidence instead of fear.

Related to this, Hull and colleagues [85] claimed for a “drinking water microbiome project”, highlighting the crucial role of all the possible stakeholders, from the academic to the water companies and to the civil society.

This is in line with the principle of a responsible research and innovation (RRI) action, which can be the driver for building a cultural shift.

A deeper knowledge on drinking water microbiome and an effective information of citizens about water quality will help in gaining awareness on how water with its biodiversity is a valuable resource to be preserved. Moreover, increased confidence about tap water quality can lead to positive side effects, such as the reduction in unnecessary plastic consumption due to plastic bottles of mineral waters, limiting environmental damage.

**Figure 2:**
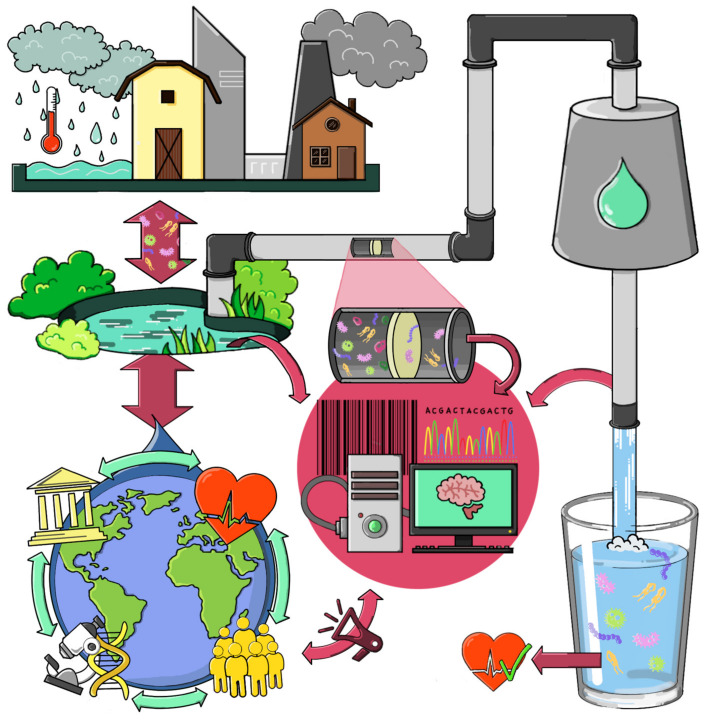
The journey of drinking water source and its microbial communities across drinking water treatment and distribution can be deciphered through omic approaches. Drinking water quality can be preserved and improved thanks to microbiome-based research, and this has multiple effects on human wellbeing, society, science, and the Earth as a whole. Bi-directional arrows highlight the mutual effect and the relations of interdependence.

Omics approaches can significantly contribute in building research that is innovative and responsible, helping in deciphering the journey of a drinking water source and its microbial communities across drinking water treatment and distribution [47,84,85]. Drinking water quality can be preserved and improved thanks to microbiome-based research, and this has multiple effects on human wellbeing, society, science, and the Earth on the whole (Figure 2).

A decisive impulse towards a more efficient management of drinking water quality is provided by the recent recommendations of Water Safety Plan based on the “WHO Guidelines for drinking water quality”: it promotes a proactive approach, moving towards risk prevention through good management of the complete water supply system, instead of simply monitoring indicator parameters.

Such a mission collimates with a One Health approach, defined as “*an integrated, unifying approach that aims to sustainably balance and optimize the health of people, animals and ecosystems. It recognizes the health of humans, domestic and wild animals, plants, and the wider environment (including ecosystems) are closely linked and inter-dependent*” [86]. This vision aims at encompassing the interconnections across habitats and human–nature interactions. Such a comprehensive perspective was already recommended, for instance, to defeat antibiotic resistance in wastewater environments [87], highlighting the challenges of integrating science with societal needs, but also the necessity of looking from a new angle at past and upcoming environmental issues. Furthermore, the One Health approach was broadened proposing a Global Health approach [88], which extends the concept to a worldwide view, integrating actions taken by countries, international organizations and other actors involved at the global scale.

## 7. Conclusions

The studies discussed here depict a compelling picture of the complex interactions among all the players shaping the equilibrium of drinking water microbiome, spanning from the unseen majority of environmental microorganisms to the bioindicators species. Humans must be considered actors as well.

What clearly appears is that biological systems are more complex than we can imagine, and not every dynamic can be explained taking into account only specific indicator parameters. Bacteria, with their extremely diversified metabolic capacities, evolutionary adaptivity and the aptitude to respond to external stimuli, are the key players in water ecosystem health. In this respect, omics technologies will contribute to remarkable advances not only in water systems but also in microbiome science as a whole.

A natural progression in biomonitoring strategy will comprehend a framework for the comprehensive analysis of drinking water microbiome to derive significant information about microbial dynamics sustaining ecosystem health and resilience to anthropogenic stressors.

A large-scale coordinated action providing for shared funding schemes,, involvement of laboratories around the world, and cooperation with utilities and the society is now decisive.

## Data Availability

All the data are reported in Supplementary Materials.

## Supplementary Materials

The following supporting information can be downloaded at: https://www.mdpi.com/article/10.3390/ijerph19137940/s1, Table S1: ISI Web of Science drinking water microbiome papers collection. Figure S1: Abstract texts word cloud.

## Abbreviations

List of Abbreviations and Acronyms:

BE	Built Environment
CP	Candidate Phyla
CPR	Candidate Phyla Radiation
DPANN	Diapherotrites, Parvarchaeota, Aenigmarchaeota, Nanoarchaeota, and Nanohaloarchaeota
DWDS	Drinking Water Distribution System
DWTP	Drinking Water Treatment Plan
GAC	Granular Activated Carbon
HTS	High-Throughput Sequencing
NGS	Next Generation Sequencing
NOM	Natural Organic Matter
RRI	Responsible Research and Innovation
SDG	Sustainable Development Goal
UWWTP	Urban wastewater treatment plant
