# Supplementary material for: It’s a Long Way to the Tap: Microbiome and DNA-Based Omics at the Core of Drinking Water Quality
Source: Int J Environ Res Public Health. 2022 Jun 28;19(13):7940. doi: 10.3390/ijerph19137940 (PMC9266242; doi:10.3390/ijerph19137940)
Supplement: Supplementary file 1 [file ijerph-19-07940-s001.zip › ijerph-1729745-supplementary/Figure S1.pdf]

Supplementary Material

It's a long way to the tap: microbiome and DNA-based omics at the core of drinking water quality

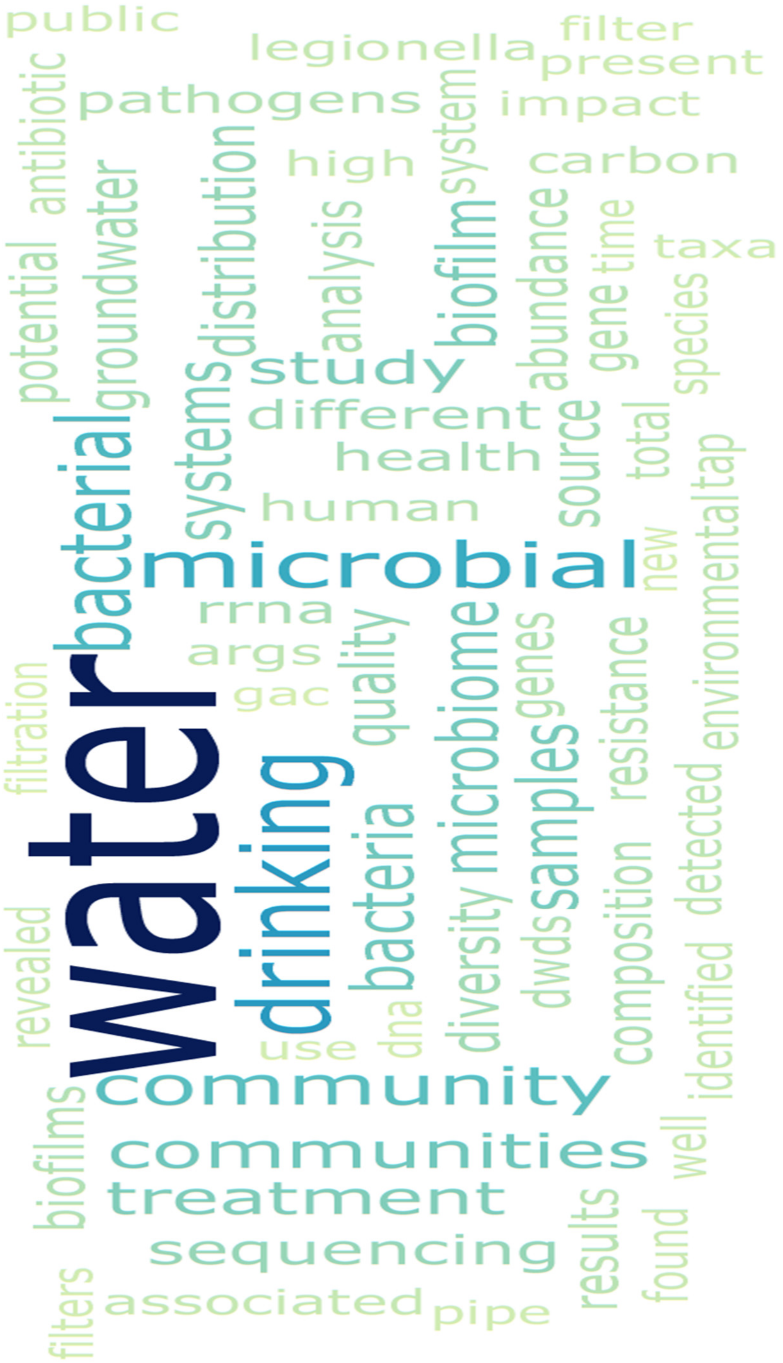

Figure S1. Abstract texts word cloud. Word cloud was built starting from abstract texts of Supplementary Table S01 using Lexos (http://lexos.wheatoncollege.edu/). The count range displayed spans from the word "water"(the most abundant, count: 1177) to "gac" (count: 44); "diversity" counts 111. "Legionella" is the only term referring to a taxon that appears from the text analysis (count: 66).
